# The structure of a purple acid phosphatase involved in plant growth and pathogen defence exhibits a novel immunoglobulin-like fold

**DOI:** 10.1107/S205225251400400X

**Published:** 2014-02-28

**Authors:** Svetlana Vladimirovna Antonyuk, Mariusz Olczak, Teresa Olczak, Justyna Ciuraszkiewicz, Richard William Strange

**Affiliations:** a Molecular Biophysics Group, Faculty of Health and Life Sciences, University of Liverpool, Crown Street, Liverpool L69 7ZB, England; b Laboratory of Biochemistry, Faculty of Biotechnology, University of Wroclaw, F. Joliot-Curie 14A, 50-383 Wroclaw, Poland

**Keywords:** purple acid phosphatase, diphosphonucleotide phosphatase, phosphodiesterase, PPD1, bimetallic Fe–Mn, fibronectin type III domain, crystal structure, SAXS

## Abstract

Phosphatases function in the production, transport and recycling of inorganic phosphorus, which is crucial for cellular metabolism and bioenergetics, as well as in bacterial killing, since they are able to generate reactive oxygen species *via* Fenton chemistry. Diphosphonucleotide phosphatase/phosphodiesterase (PPD1), a glycoprotein plant purple acid phosphatase (PAP) from yellow lupin seeds, contains a bimetallic Fe–Mn catalytic site which is most active at acidic pH. Unlike other plant PAPs, PPD1 cleaves the pyrophosphate bond in diphosphonucleotides and the phosphodiester bond in various phosphodiesters. The homohexameric organization of PPD1, as revealed by a 1.65 Å resolution crystal structure and confirmed by solution X-ray scattering, is unique among plant PAPs, for which only homodimers have previously been reported. A phosphate anion is bound in a bidentate fashion at the active site, bridging the Fe and Mn atoms in a binding mode similar to that previously reported for sweet potato PAP, which suggests that common features occur in their catalytic mechanisms. The N-terminal domain of PPD1 has an unexpected and unique fibronectin type III-like fold that is absent in other plant PAPs. Here, the *in vitro* DNA-cleavage activity of PPD1 is demonstrated and it is proposed that the fibronectin III-like domain, which ‘overhangs’ the active site, is involved in DNA selectivity, binding and activation. The degradation of DNA by PPD1 implies a role for PPD1 in plant growth and repair and in pathogen defence.

## Abstract 2

The plant purple acid phosphatase PPD1 forms a novel hexameric structure with a fibronectin III-like domain that is involved in DNA selectivity, binding and activation. The degradation of DNA by PPD1 implies a role for PPD1 in plant growth and repair and in pathogen defence.

## 1. Introduction   

Purple acid phosphatases (PAPs; EC 3.1.3.2) catalyze the hydrolysis of inorganic phosphorus from a broad range of phosphate monoesters and anhydrides in the pH range 4–7 (Olczak *et al.*, 2003 ▶). The enzymes function in the production, transport and recycling of inorganic phosphorus, which is crucial for cellular metabolism and bioenergetics, as well as in bacterial killing, since they are able to generate reactive oxygen species *via* Fenton chemistry. PAPs are acidic metallohydrolases with the metal centre comprising an iron(III) ion and a divalent metal, which is either a zinc(II), manganese(II) or iron(II) ion. The active site comprises a characteristic set of seven highly conserved amino-acid residues binding to the dinuclear metal centre. The iron(III) ion coordinates to tyrosine, histidine and aspartate residues and a hydroxo/aqua ligand, while the divalent metal coordinates to two histidine residues, an asparagine and a terminal aqua ligand; an aspartate residue bridges the two metal ions (Schenk *et al.*, 2005 ▶, 2008 ▶; Boudalis *et al.*, 2007 ▶; Klabunde *et al.*, 1995 ▶).

Eukaryotic PAPs have been classified into two main groups: high-molecular-weight (∼55 kDa) and low-molecular-weight (∼35 kDa) enzymes (Flanagan *et al.*, 2006 ▶). Multiple PAP-like isoforms have been identified in the genomes of *Arabidopsis thaliana* (Schenk, Guddat *et al.*, 2000 ▶), sweet potato (Schenk *et al.*, 1999 ▶; Durmus *et al.*, 1999 ▶), tomato (Bozzo *et al.*, 2002 ▶), soybean (Schenk *et al.*, 1999 ▶), red kidney bean (Schenk *et al.*, 1999 ▶) and potato (Zimmermann *et al.*, 2004 ▶) and in prokary­otic genomes (Schenk, Korsinczky *et al.*, 2000 ▶). The best characterized enzymes among plant PAPs are the Fe–­Zn phosphatase purified from red kidney bean (*Phaseolus vulgaris*; Klabunde *et al.*, 1995 ▶, 1996 ▶; Sträter *et al.*, 1995 ▶) and the Fe–Mn-containing sweet potato PAP, which has an increased catalytic activity for phosphate esters and has been shown to require manganese(II) (Schenk *et al.*, 2005 ▶).

Recently, we identified, purified and characterized a diphosphonucleotide phosphatase/phosphodiesterase (PPD1) from yellow lupin (*Lupinus luteus*) seeds, which belongs to a novel group of high-molecular-weight (75 kDa) plant PAPs with one Fe atom and one Mn atom (1:1 molar ratio) per subunit (Olczak *et al.*, 2000 ▶, 2009 ▶; Olczak & Watorek, 2000 ▶; Olczak & Olczak, 2005 ▶). The enzyme is a glycoprotein, possessing paucimannosidic, high-mannose and complex-type oligosaccharides (Olczak *et al.*, 2000 ▶). Among the complex-type N-­glycans, Lewis epitope structures have been found which are characteristic of extracellular plant proteins. However, the presence of paucinomannosidic N-glycans might suggest vacuolar location of PPD1. Unlike typical PAPs, PPD1 cleaves the pyrophosphate bond in diphosphonucleotides and the phosphodiester bond in various phosphodiesters, and has a high affinity towards the diphosphate bond in organic and inorganic pyrophosphates, with the highest specificity towards diphosphonucleotides (Olczak *et al.*, 2000 ▶). Its substrate specificity is similar to that of nucleotide pyrophosphatase/phosphodiesterase from soybean leaves (Salvucci & Crafts-Brandner, 1995 ▶), with the exception of its low affinity towards nucleotide-sugars (GDP-glucose and UDP-glucose). PPD1 cleaves the pyrophosphate bond but differs from plant soluble pyrophosphatases in its preference to act at slightly acidic pH.

Here, we report the solution and single-crystal X-ray structures of PPD1 for the first time, revealing that it possesses an immunoglobulin fold in the N-terminal domain and functions as a homohexamer, features that are both unique among PAPs characterized to date.

## 2. Methods and materials   

### 2.1. Purification of PPD1   

The purification of native PPD1 from yellow lupin seeds and determination of its enzymatic activity were performed as described previously (Olczak & Watorek, 1998 ▶; Olczak *et al.*, 2009 ▶).

### 2.2. Analysis of DNase activity   

The ability of PPD1 to cleave DNA was examined by following the digestion of plasmid DNA and linear lambda DNA. Digestion products were separated using agarose-gel electrophoresis. Circular plasmid DNA (p3XFLAG-CMV-26; Sigma) at a concentration of 22 ng µl^−1^ in 20 m*M* MES buffer pH 6.0 containing 0.2 *M* NaCl and 0.1% Triton X-100 was treated with PPD1 (50 ng µl^−1^) at 37°C. Subsequently, 20 µl aliquots were removed and reaction was stopped by heating at 90°C for 10 min. A similar procedure was employed for linear high-molecular-weight lambda DNA (Sigma), with the exception that DNA was used at a concentration of 20 ng µl^−1^ and PPD1 at a concentration of 100 ng µl^−1^. Samples were subjected to electrophoresis on 1% or 0.6% agarose gels containing ethidium bromide in 1× TAE buffer at 100 V for approximately 1 h. The resulting gels were visualized using the GelDoc documentation system (Bio-Rad). A tube test for DNase activity was performed according to Kunitz (1950 ▶) with modifications. Briefly, the reaction was carried out continuously at 25°C in 1.2 ml 0.1 *M* acetate buffer pH 5.0 containing 0.85% NaCl and 10 m*M* MgSO_4_, using 0.0033% DNA from calf thymus (Sigma), and the decrease in absorbance at 260 nm was measured (Beckman DU-640). Standardized DNase I from bovine pancreas (4000 Kunitz units per milligram; Sigma) was used as a control. Samples were analyzed in triplicate. DNase-free chemicals and sterile water were used in all experiments.

### 2.3. Crystallization   

Crystals were grown using the hanging-drop method at room temperature by equilibration of 2 µl 5 mg ml^−1^ PPD1 solution in 20 m*M* sodium acetate buffer, 0.5 *M* NaCl at pH 5.2 with 2 µl ready-made solution No. 39 (box 1) from the Morpheus screen (Molecular Dimensions) over 100 µl of the same Morpheus reservoir solution. Solution 39/1 consists of 0.12 *M* Alcohols Mix (equal composition of 1-butanol, 2-­propanol, 1,3-propanediol, 1,2-propanediol, 1,4-butanediol and 1,6-hexanediol), 0.1 *M* Buffer System 1 (1 *M* MES and 1 *M* imidazole) at pH 6.5 and 30% precipitant comprising 40% glycerol and 20% PEG 4000. Crystals were cryocooled in reservoir solution and stored in liquid nitrogen prior to data collection.

### 2.4. X-ray data collection, processing and structure determination   

This is described in full in the Supporting Information. The PROXIMA1 beamline at SOLEIL equipped with an ADSC Q315 3 × 3 CCD detector was used to collect the crystallo­graphic data from PPD1 single crystals. The small-angle X-ray scattering (SAXS) data for PPD1 in solution (1–4 mg ml^−1^ concentration) were collected using the SWING beamline at SOLEIL.

## 3. Results and discussion   

### 3.1. Overall structure of PPD1   

**Figure 1:**
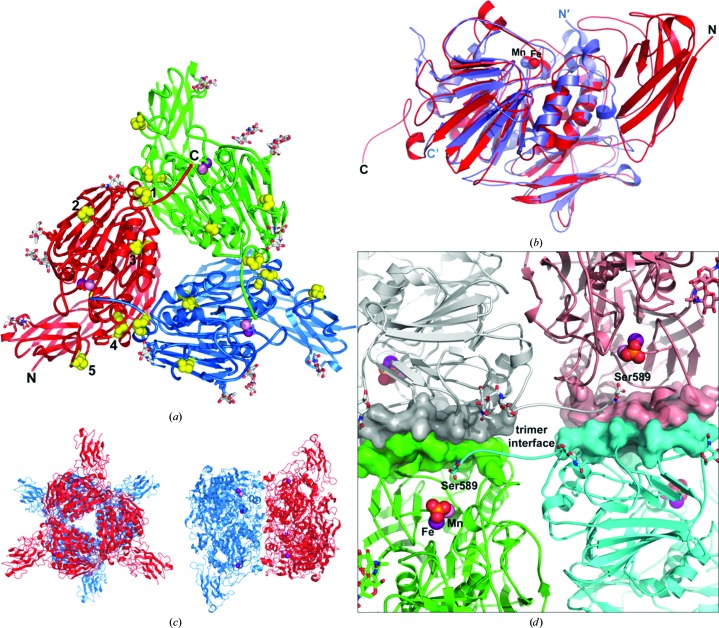
The crystal structure of PPD1. (*a*) The PPD1 asymmetric unit is a trimer, shown as a cartoon coloured by individual subunits, with their N- and C-­terminal ends indicated. The metal atoms in each subunit are shown as pink (Mn) and purple (Fe) spheres, with the bound phosphate ligands depicted as sticks. The active sites are 54 Å apart. Disulfides are shown as yellow spheres and sugar groups as silver and red sticks; there are five of each per subunit (one is occluded in the figure). The cysteines or disulfide bridges labelled **1** (Cys577–Cys582), **2** (Cys484–Cys491) and **3** (Cys396–Cys417) constrain flexible loops and help to position the interfacing residues, while that labelled **4** (Cys202–Cys366) is involved in hydrogen bonding to the adjacent subunit. Disulfide bridge **5** (Cys69–Cys82) bridges an extended loop (or turn) in the N-terminal domain and orients it towards the solvent channel above the active site. The sugars are covalently bound to Asn residues 92, 241, 292, 502 and 525. For clarity, water molecules have been omitted. (*b*) Cartoon representation of the superposition, using secondary-structure matching (*SSM*) in three dimensions (Krissinel & Henrick, 2004*a* ▶), of PPD1 (red) with a sweet potato PAP (blue) subunit taken from PDB entry 1xzw (Schenk *et al.*, 2005 ▶). The metal atoms are shown as spheres. The alignment of 353 residues gave an overall r.m.s.d. of 1.58 Å between the two structures, with a *Z*-score of 17.9, and identified the extent of the PAP domain. The C-­terminal end of PPD1, which is 19 residues longer than in sweet potato PAP, extends into the active-site cavity of the neighbouring subunit of the trimer. The fibronectin type III domain identified by Tsyguelnaia & Doolittle (1998 ▶) for red kidney bean PAP is also present in sweet potato and PPD1, and is located at the lower centre of the figure. The N-terminal end comprising residues 1–150 is unique to PPD1 and is not part of the known PAP family. (*c*) The PPD1 hexamer generated from crystallographic symmetry, showing views along and perpendicular to the trimeric axis. (*d*) The trimer–trimer interface. The active sites are positioned in the interior of the hexamer, approximately 38 Å apart, with 12 intersubunit hydrogen-bonding interactions located at each of the interfaces, directly between the active sites. The residues involved in hydrogen bonding are represented by transparent surfaces, while the active sites (with bound phosphate) are shown as spheres. Sugar groups are shown as sticks. The C-terminal ends of the silver and cyan subunits, belonging to separate trimers, traverse the interface region in the solvent-filled channels and are shown with their terminal Ser589 (stick) residues poised above the bound phosphate groups.

Previously, we had predicted a three-dimensional model from the PPD1 sequence using the structure of sweet potato PAP (PDB entry 1xzw) as a template (Olczak *et al.*, 2009 ▶). This model included only the sequence 158–602 of PPD1, *i.e* its N-­terminal sequence was omitted. The crystal structure reported here confirms the accuracy of the predicted homology model for this segment of the protein. The overall structure of the PPD1 subunit is defined by two domains: a sweet potato-like PAP domain, containing the Fe–Mn active site, and a previously uncharacterized N-terminal domain with unknown function. The crystallographic asymmetric unit contains three identical subunits (polypeptide chains *A*, *B* and *C*), which together form a homotrimer (Fig. 1 ▶ *a*). Five disulfide bridges are present within each subunit, three of which are involved in positioning the structural elements that form the trimer interfaces. There are no intersubunit disulfides involved in forming the PPD1 trimer or hexamer. A possible 22 hydrogen bonds and eight salt bridges between each of the adjacent subunits help to stabilize the trimeric assembly, with 7% of the solvent-accessible surface area buried at each intersubunit interface. The solvent-accessible C-terminal end of each subunit (residues 583–589) is oriented by the Cys577–Cys582 bridge so that it traverses the adjoining subunit, protruding into its Fe–Mn active site and placing the side chain of the terminal Ser589 within 8 Å of the two metal atoms. The N-terminal region (residues 1–150) is longer than in other PAPs and has very low sequence homology to target protein sequences in UniProt and other protein-sequence databases, yielding no identifiable or conserved domains. However, the remainder of the PPD1 structure up to the C-terminal end has 24% sequence identity to sweet potato PAP (Olczak *et al.*, 2009 ▶) and is structurally very similar, with an r.m.s.d. of 1.58 Å when aligned using *SSM* superposition (Krissinel & Henrick, 2004*a* ▶; Fig. 1 ▶ *b*). There are no disulfides in the sweet potato PAP subunit, but its biological dimer is linked by an intersubunit disulfide bridge (there is no equivalent subunit interface bridge in PPD1) located on extended loops. The equivalent loops in each PPD1 subunit are forced by a disulfide bridge (Cys484–Cys491; Fig. 1 ▶ *a*) to adopt a different orientation to that in sweet potato PAP. This structural constraint in PPD1 serves to create space for the C-­terminal end of one subunit to contact the active site of the adjacent subunit, as described above.

### 3.2. The oligomeric state and biological unit of PPD1   

Unlike mammalian PAPs, which function as monomers, the plant enzymes have been described as homodimeric glycoproteins with a molecular weight of ∼110 kDa. Our previously published data on native and recombinant PPD1 shows that while the glycosylation state influences protein migration during gel-filtration chromatography, there is clear evidence for purified PPD1 forming higher oligomers (Olczak *et al.*, 2000 ▶; Olczak & Olczak, 2005 ▶). Analysis of the crystal structure of PPD1 using the *PDBePISA* server (Krissinel & Henrick, 2007 ▶) suggests that its most probable quartenary structure is a hexamer, with an estimated dissociation free energy of 42 kcal mol^−1^ compared with 24 kcal mol^−1^ for the trimer. The hexamer generated from crystallographic symmetry (Fig. 1 ▶ *c*) possesses a trimer–trimer interface that includes 36 hydrogen bonds (12 symmetry-related hydrogen bonds per subunit–subunit interaction) and which buries 12% of the solvent-accessible surface area. The trimers are rotated by approximately 60° with respect to each other, giving a staggered appearance to the N-terminal domains when viewed along the threefold axis. The stabilizing trimer–trimer hydrogen bonds are all contained at the interfaces between the active sites, which are separated by 38 Å and are fully solvent-exposed (Fig. 1 ▶ *d*). A sugar molecule is also bound to each subunit at this region of the interface, to Asn525. The symmetry of the hexamer entails that the C-terminal loops of adjacent subunits from each trimer are extended into the solvent channel and separated from each other by approximately 8–10 Å across the trimer–trimer interface. The concentration of hydrogen bonds at the interface between the two active sites, along with the positioning of the C-terminal ends in the hexamer and their extension into the active sites in each trimer to within 8 Å of the metals, argue that these structural elements are involved in allosteric regulation of the catalytic activity.

**Figure 2:**
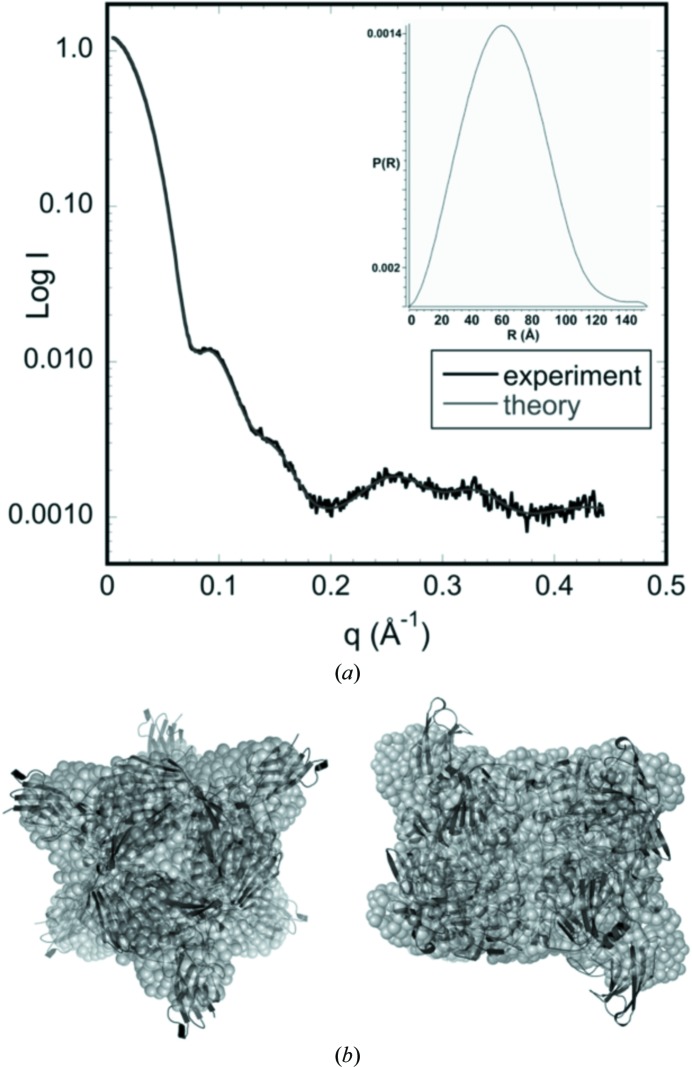
Small-angle X-ray scattering (SAXS) of PPD1. (*a*) The experimental spectrum is shown with a simulated fit obtained using *GASBOR*. The radius of gyration, *R*_g_, was estimated from the low-angle scattering region using a Guinier plot, *i.e.* log(intensity) *versus* *q* ^2^. The distance-distribution function, *P*(*r*), with maximum linear dimension *D*_max_ = 159.4 Å, is shown in the insert. (*b*) The *ab initio* shape reconstruction by *GASBOR* using *P*3_2_ symmetry, showing agreement between the predicted molecular shape (grey beads) and the hexameric construct based on the crystal structure (black cartoon). Water molecules have been omitted.

Experimental confirmation of the hexameric form of PPD1 in the solution state was obtained by SAXS (small-angle X-ray scattering) measurements (Fig. 2 ▶ *a*). The radius of gyration, *R*_g_, calculated from the Guinier region of the SAXS data is 49.8 ± 0.1 Å. The *R*_g_ calculated from the crystal structure for the trimer is 37 Å, while the *R*_g_ calculated from the crystallo­graphic symmetry hexamer is 46 Å. A fit of the crystallo­graphic hexamer to the SAXS spectrum using *CRYSOL* (Svergun *et al.*, 1995 ▶) gives a χ of 2.5 compared with 51 for the trimer model. In these calculations, the contribution to the scattering from the covalently bound sugars was not taken into account. Shape reconstructions from the solution SAXS data (Fig. 2 ▶ *b* and Supplementary Fig. S1) are consistent with the crystallographic hexamer, providing convincing evidence that this is the genuine oligomeric state of PPD1 and is not the result of crystal packing.

A homohexameric quaternary structure is unique for a PAP, since previously only homodimers have been reported for the plant enzymes. Among similar enzymes which do function as hexamers are the soluble pyrophosphatases (PPases), which hydrolyse inorganic pyrophosphate to two orthophosphates. All soluble PPases are homo-oligomers: dimers in eukaryotes and hexamers or tetramers in prokayotes. The best characterized PPases are those from *Escherichia coli* and *Saccharomyces cerevisiae* (Cooperman *et al.*, 1992 ▶; Baykov *et al.*, 1999 ▶). The biological unit of *E. coli* PPase is formed by six identical subunits of 20 kDa each arranged with *D*_3_ symmetry in two layers of trimers (*i.e.* a dimer of two trimers; Kankare *et al.*, 1996 ▶; Harutyunyan *et al.*, 1997 ▶). However, PPD1 and the PPases are different in their activities and show no sequence and structural homology.

### 3.3. The Fe–Mn active site   

**Figure 3:**
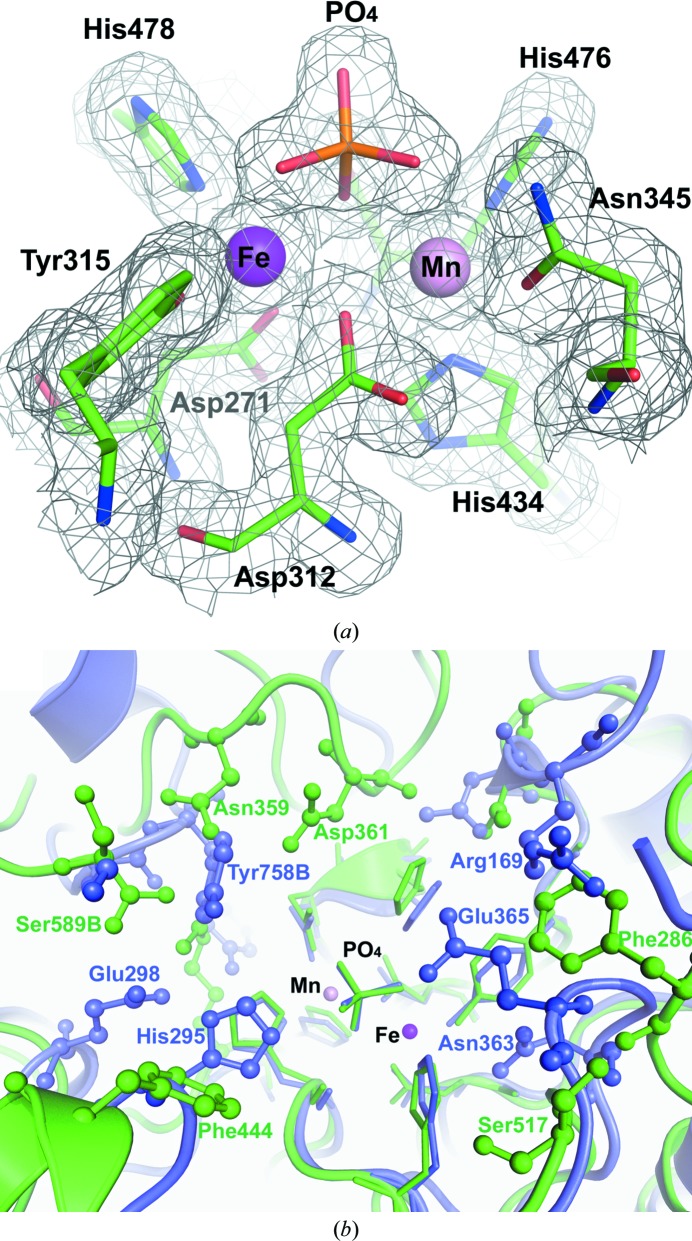
(*a*) The active site of PPD1 with bound phosphate. The Fe and Mn atoms are shown as purple and pink spheres, respectively. The average metal–protein ligand distances for the three subunits are Tyr315 1.9 Å, His478 2.2 Å, Asp312 2.3 Å and Asp271 2.1 Å at the Fe site, and His434 2.2 Å, His476 2.2 Å, Asn345 2.1 Å and Asp312 2.3 Å at the Mn site. The phosphate anion is coordinated to both metals *via* a bridging O atom (Fe/Mn—O distance of ∼2.3 Å). The sixth coordination position of each metal is provided by two of the remaining phosphate O atoms at ∼2.5 Å. The 2*F*_o_ − *F*_c_ electron-density maps are shown contoured at 1.2σ. (*b*) The active site of PPD1 (green) and sweet potato PAP (blue). A view is shown from the molecular surface looking into the bimetallic centre, with the conserved metal-ligand residues represented by sticks and the neighbouring, largely nonconserved, active-site residues shown in ball-and-stick representation. The substrate-entry space is larger and more hydrophobic in PPD1 (*e.g.* Phe286 and Phe444 at the substrate-entry pocket are separated by ∼12 Å, compared with the ∼6 Å separation between His295 and Glu365 in sweet potato PAP), which allows significant differences in substrate accommodation and substrate reactivity (*e.g.* with bis-*p*NPP).

PAPs from different sources, including PPD1, show a high degree of conservation among the residues present in the catalytic centre (Klabunde *et al.*, 1995 ▶; Guddat *et al.*, 1999 ▶; Lindqvist *et al.*, 1999 ▶; Uppenberg *et al.*, 1999 ▶; Schenk *et al.*, 2005 ▶; Sträter *et al.*, 2005 ▶). Previously, we identified an Fe–Mn bimetallic centre in PPD1, examined its oxidation state and demonstrated that it requires iron and divalent metal for its enzymatic activity (Olczak *et al.*, 2009 ▶). The crystal structure shows the active site of PPD1 with a phosphate ion bound to both of the metal atoms, similar to that previously described to be unique for sweet potato PAP (Schenk *et al.*, 2005 ▶). Anomalous scattering peaks confirm the identities of the metals at the active site (Supplementary Fig. S2). The coordination environment of the metals is identical in each of the three subunits of the trimer. The Fe atom is coordinated by protein residues His478, Tyr315, Asp271 and Asp312, which bridges the two metal atoms (Fig. 3 ▶ *a*). The Mn atom is coordinated by His434, His476, Asn345 and the bridging Asp312. One O atom of the bound phosphate anion acts as a bridge between the two metal ions, and two more O atoms from the phosphate group complete the coordination spheres of both metals. Although the exact process is still under debate, a general model of the catalytic mechanism of PAPs has been reported by several investigators. The detailed description has mainly been based on the crystal structures of free red kidney bean PAP and its complexes with phosphate (which is both a reaction product and a substrate analogue) and tungstate (an inhibitor) (Sträter *et al.*, 2005 ▶; Klabunde *et al.*, 1996 ▶), and on the structure of the phosphate-bound sweet potato PAP (Schenk *et al.*, 2005 ▶). The structures of red kidney bean PAP with bound sulfate and fluoride provided further insights into the pre-catalytic phase of the enzymatic reaction (Schenk *et al.*, 2008 ▶), from which an eight-step model for the reaction mechanism was proposed. Briefly, in the initial step of the catalytic cycle the phosphate group is bound to the divalent metal ion, displacing a water molecule (Schenk *et al.*, 2008 ▶; Twitchett *et al.*, 2002 ▶). The substrate is then oriented and activated by this centre to facilitate nucleophilic attack by a hydroxide group (Boudalis *et al.*, 2007 ▶; Schenk *et al.*, 2005 ▶, 2008 ▶; Smoukov *et al.*, 2002 ▶). The iron(III)-bound hydroxide group can attack the electrophilic atom of the substrate, initiating ester-bond hydrolysis (Klabunde *et al.*, 1996 ▶; Lindqvist *et al.*, 1999 ▶; Schenk *et al.*, 2008 ▶; Uppenberg *et al.*, 1999 ▶; Merkx *et al.*, 1999 ▶). A mechanism in which the μ-hydroxide bridge acts as the nucleophile for hydrolysis of the phosphate has been also proposed (Schenk *et al.*, 2008 ▶; Smoukov *et al.*, 2002 ▶). After hydrolysis of the substrate, the phosphate is bound to the PAP metal centre (Guddat *et al.*, 1999 ▶; Schenk *et al.*, 2005 ▶). While the active-site structure of PPD1 fits into this general scheme, the physiological roles and specific substrates of different PAPs still have to be determined and modifications to this mechanism are anticipated. While the majority of PAPs do not exhibit the ability to hydrolyze diesters, based on their lack of activity with bis(*p*-nitrophenyl) phosphate (bis-*p*NPP), PPD1 does hydrolyse diesters (Olczak *et al.*, 2000 ▶; Olczak & Olczak, 2002 ▶), behaviour that is similar to model complexes designed to mimic the PAP metal centre (Olczak & Watorek, 1998 ▶; Xavier *et al.*, 2009 ▶). This difference in substrate selectivity and reactivity of the enzymes probably results from the amount of steric crowding (Cox *et al.*, 2007 ▶) and degree of hydrophobicity of the active sites. This is indicated, for example, by comparison between the sweet potato PAP and PPD1 active sites, which shows that the bimetallic site in the latter is more accessible and open and contains more hydrophobic residues (Fig. 3 ▶ *b*). In sweet potato PAP, substrate orientation, specificity and transition-state activation involve residues His295 and Glu365, both of which form a hydrogen bond to the uncoordinated O atom of the phosphate anion (Schenk *et al.*, 2005 ▶). In PPD1, both of these residues are substituted positionally by Phe residues, which are located ∼5 Å further away from the binding site of the phosphate anion. The phosphate O atom is instead hydrogen-bonded to one of several water molecules that occupy the vacant space left by the larger active-site cavity. A different catalytic model and an alternative substrate are required for PPD1 compared with sweet potato PAP, and further investigation is needed to clarify these details.

### 3.4. N-terminal domain structure and topology   

**Figure 4:**
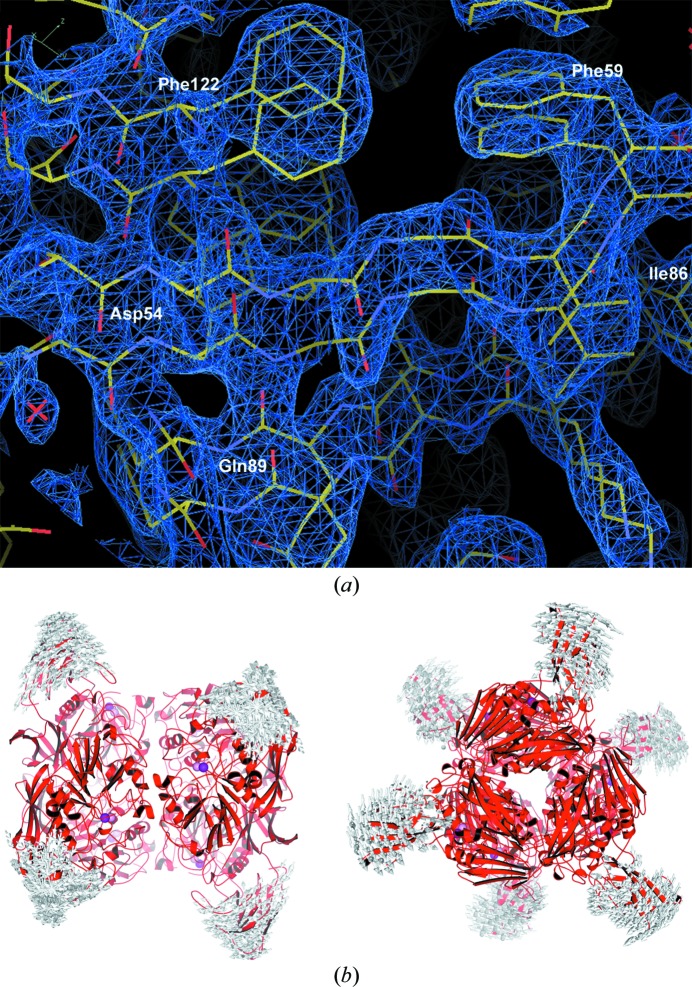
(*a*) The 2*F*_o_ − *F*_c_ electron-density map (contoured at 1σ) of the portion of the N-terminal domain of the PPD1 subunit with the highest overall *B* factor, showing evidence for two main chains in this region of the structure. (*b*) Graphical depiction of all-atom normal-mode states of the PPD1 hexamer. The trajectories for the first six nontrivial low-frequency normal modes are shown superimposed as directional arrows. The largest structural fluctuations are centred on the N-terminal domains, which undergo rigid-body displacements relative to the PAP domain. Determination of the motional correlation between these domain fluctuations and their import with respect to potential allosteric properties would require the analysis of a larger number of nodes (Wynsberghe & Cui, 2006 ▶); nevertheless, these low-frequency modes may have a functional significance in view of the proximity of the N-terminal domains to the active sites, shown here as pink (Mn) and purple (Fe) spheres.

Genes encoding PAPs have been found in mammalian, plant, mycobacterial and fungal genomes, but the phylogeny of PAPs is still a matter of debate. It has been speculated that the catalytic domains of PAPs may have evolved through the combination of mononuclear centres (Guddat *et al.*, 1999 ▶). However, the origin of the noncatalytic N-terminal domain present in some PAPs remains unknown and does not preclude a convergent type of evolution. Structural evidence for the flexibility of the entire N-terminal end of the protein is provided by its higher average C^α^-atom *B* factor (26 Å^2^) compared with the PAP domain (18 Å^2^) and by the presence of two main-chain conformations, comprising 120 residues of the domain, that are clearly traceable in the electron-density maps of one of the three subunits of each trimer (Fig. 4 ▶ *a*). A normal-mode analysis of the hexamer, performed using the *NOMAD-Ref* server (Lindahl *et al.*, 2006 ▶), shows that the first six nontrivial low-frequency modes are dominated by the movement of this domain relative to the PAP domain, which remains relatively fixed (Fig. 4 ▶ *b*). Since the N-terminal domain ‘overhangs’ the PAP domain, with its extended loop fluctuating in the solvent channel of the active site during these movements, such structural transitions may have a functional importance during catalysis.

**Figure 5:**
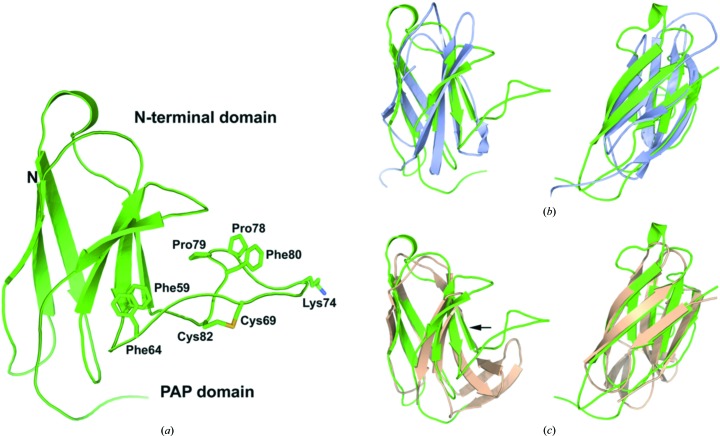
(*a*) The N-terminal domain of each PPD1 subunit comprises two β-sheets formed from seven β-strands and encompasses residues 1–150. The first 20 residues were not modelled in the structure as they were not visible in the electron-density maps. (*b*) The PPD1 N-terminal end (green) aligned with the structure of the fibronectin type III domain of human sidekick-2 (PDB entry 1wfn; *Z* = 4.6, r.m.s.d. = 2.99 Å, 76 aligned residues, chain *A*), an s-type immunoglobulin. The same orientation as in (*a*) is shown in the left panel and that with the view rotated about the vertical axis by 90° is shown in the right panel. A portion of the N-terminal loop of PDB entry 1wfn occluding the view has been omitted for clarity. (*c*) The same viewpoints are shown for the PPD1 N-terminal end (green) aligned with the structure of the antitumour antibiotic C-1027 apoprotein (PDB entry 1j48; *Z* = 5.9, r.m.s.d. = 2.04 Å, 80 aligned residues, chain *A*), which is a v-type immunoglobulin. The seven β-strands in PPD1 all have corresponding well aligned counterparts in the nine-stranded 1j48 structure. The ‘extra’ hairpin β-strands are replaced by a short loop in PPD1. The enediyne chromophore in the holo form of C-1027 is bound at a hydrophobic pocket, the position of which is indicated by the arrow.

The N-terminal domain (residues 1–150) of PPD1 consists primarily of two β-sheets making up a seven-stranded β-­barrel. A 26-residue loop (Phe59–Pro85) joining β-strands 3 and 4 extends about 20 Å from the barrel and into the active-site solvent channel of the PAP domain. This extension positions the Lys74 side chain on the loop 10 Å from the phosphate-binding site and 8 Å from the Ser589 residue of the neighbouring subunit. The loop is stabilized by a Cys69–Cys82 disulfide bridge and a Pro78-*cis*-Pro79-Phe80 motif, with a π-­stacking interaction between Pro78 and Phe80 and additional π-stacking between Phe59 and Phe64 (Fig. 5 ▶ *a*).

Structural homologues of the N-terminal domain were found by searching the PDB using the *SSM* (*PDBeFold*) server (Krissinel & Henrick, 2004*b* ▶). The top hits, all with *Z*-­scores of <6 and *Q*-scores of <0.3, were found for proteins containing s-type or v-type immunoglobulin-like folds. These included the fibronectin type III (FN3) domain of human sidekick-2, the DNA-binding antitumour antibiotic chromoprotein C-1027 (Tanaka *et al.*, 2001 ▶) and the human T-cell surface glycoprotein CD8 alpha chain (Gao *et al.*, 1997 ▶). The seven-stranded β-barrel arrangement of PPD1 is consistent with an s-type immunoglobulin fold (Bork *et al.*, 1994 ▶; Fig. 5 ▶ *b*). However, the PPD1 structure lacks the strongly conserved disulfide bridge and tryptophan residue characteristically found at the core of the β-barrel (Ioerger *et al.*, 1999 ▶). The structural similarity of the N-terminal domain of PPD1 to the apo form of the antitumour antibiotic protein C-1027 and related chromoproteins (*e.g.* neocarzinostatin and kedarcidin) is intriguing in view of their DNA-cleaving properties when complexed with enediyne chromophores. C-1027 has a v-type immunoglobulin fold and gave the best overall score for the structural alignment with PPD1. The two hairpin β-­strands in the v-type topology are replaced in PPD1 by a short loop connecting β-strands 5 and 6 (Fig. 5 ▶ *c*). The largest structural difference between these proteins and PPD1, and excluded from the *SSM* alignment, is the much shorter loop (7–10 residues compared with 26 residues in PPD1) connecting β-­strands 3 and 4. The enediyne chromophore-binding site is located here in the C-1027 holoenzyme and is thought to be the site where its interaction with DNA occurs (Tanaka *et al.*, 2001 ▶).

FN3 domains are most common in animal proteins, having a number of important functions, including cell-surface adhesion, cell migration, blood coagulation and signalling, and they bind to a range of other molecules including DNA, heparin and collagen. FN3 domains have also been observed in a restricted set of extracellular enzymes found among a diverse set of soil bacteria (Bork & Doolittle, 1992 ▶; Hansen, 1992 ▶; Little *et al.*, 1994 ▶). In plant proteins, an FN3 type domain was first identified in the sequence of red kidney bean PAP through hidden Markov modelling (Tsyguelnaia & Doolittle, 1998 ▶) and was confirmed by the crystal structure (Sträter *et al.*, 1995 ▶). This structural motif is present in other PAPs, including PPD1 and sweet potato PAP (Fig. 1 ▶ *b*). PPD1 therefore possesses two distinct FN3-type domains, one of which is conserved in other PAPs and corresponds to residues 150–250 in the amino-acid sequence, while the second FN3 domain comprises the entire N-terminal end of the enzyme and is not present in other PAPs (see Supplementary Fig. S3). The functional significance of this latter domain is unclear, but it may be involved in substrate specificity.

### 3.5. Biological implications of PPD1   

**Figure 6:**
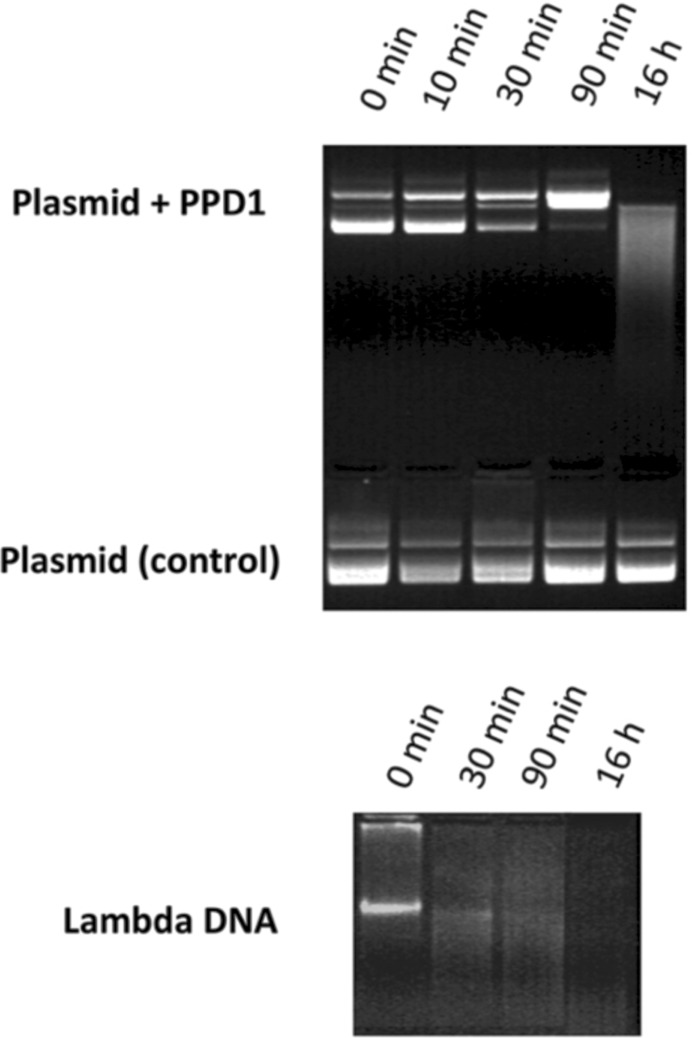
DNA-cleavage activity of PPD1. Cleavage of circular plasmid DNA (top panel) and linear high-molecular-weight lambda DNA (bottom panel) by PPD1. The digestion products were separated in 1 or 0.6% agarose gels and stained with ethidium bromide.

**Figure 7:**
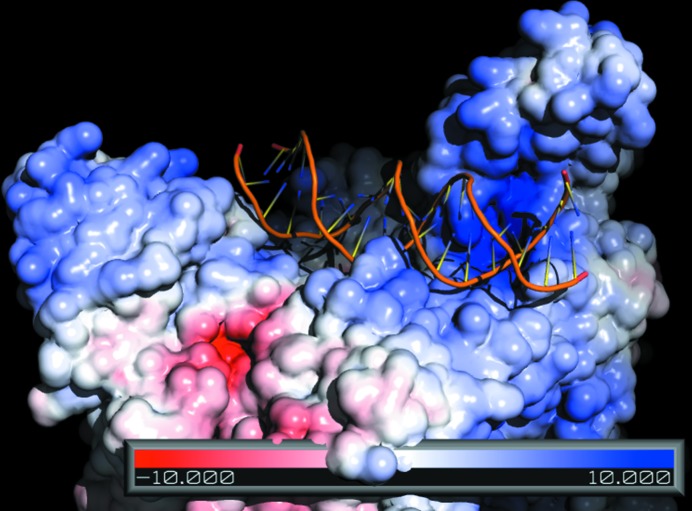
A simulated docking model of a short DNA fragment lying across the positively charged ‘groove’ on the surface of the PPD1 hexamer. The Fe–­Mn site is located at the position of maximum positive (blue) charge density. The flexible N-terminal domain of the subunit of one trimer is poised above this site and the docked DNA strand sits adjacent to it. The surface-charge distribution is shown for pH 6 in units of *kT*/e^−^. Docking calculations were performed using the program *Hex*4.5 (Ritchie & Kemp, 1999 ▶). Surface electrostatic calculations were performed using the *Adaptive Poisson–Boltzmann Solver* (*APBS*; Baker *et al.*, 2001 ▶) and were visualized using *PyMOL*. *PDB*2*PQR* (Dolinsky *et al.*, 2004 ▶) was used to prepare the coordinates for input to *APBS*.

To determine the substrate specificity of PPD1, its ability to digest and cleave circular and linear DNA was determined (Fig. 6 ▶). It has been shown in model studies that synthetic complexes that resemble the catalytic centre of PAPs with different metal compositions exhibit DNase activity, possibly occurring by intercalation (Peralta *et al.*, 2010 ▶; Lanznaster *et al.*, 2005 ▶; Xavier *et al.*, 2009 ▶). Compared with these studies, the activity of PPD1 is higher: more efficient DNA digestion was observed as examined by separation of digestion products on agarose gels. This ability of the enzyme was also confirmed by an enzymatic tube test using the standard Kunitz method (Kunitz, 1950 ▶), demonstrating a PPD1 DNase activity of 148 ± 27 Kunitz units per milligram of protein. We postulate that the FN3-type domain of PPD1 may take part in presentation of the substrate (*e.g.* DNA) to the catalytic site or in modification of the DNA structure through binding at the DNA groove, thus improving the catalytic activity of PPD1. The catalytic mechanism of PAP has been so far elaborated for monoester substrates, and phosphodiesterase activity has been demonstrated in the PAP family before, but only with small ester substrates (Cox *et al.*, 2007 ▶). In this work, we propose a DNA molecule as a potential phosphodiesterase substrate. Molecular modelling shows that DNA molecules could be bound to the surface of the PPD1 hexamer in a suitable orientation for such an interaction to occur (Fig. 7 ▶). However, at present we are unable to establish a precise catalytic mechanism, as well as a clear relationship between *in vitro* DNA cleavage and *in vivo* PPD1 function, although one may speculate that this may include degradation of DNA by PPD1 to maintain plant growth and repair or the involvement of PPD1 in pathogen defence.

## Supplementary Material

PDB reference: purple acid phosphatase, 3zk4

Supporting Information.. DOI: 10.1107/S205225251400400X/lz5001sup1.pdf
