# Supplementary material for: The structure of a purple acid phosphatase involved in plant growth and pathogen defence exhibits a novel immunoglobulin-like fold
Source: IUCrJ. 2014 Feb 28;1(Pt 2):101–9. doi: 10.1107/S205225251400400X (PMC4062093; doi:10.1107/S205225251400400X)
Supplement: Supplementary file 1 [file m-01-00101-sup1.pdf]

IUCrJ

Volume 1 (2014)

Supporting information for article:

The structure of a purple acid phosphatase involved in plant growth and pathogen defence exhibits a novel immunoglobulin-like fold

Svetlana Vladimirovna Antonyuk, Mariusz Olczak, Teresa Olczak, Justyna Ciuraszkiewicz and Richard William Strange

Supplementary Information

S1. Methods and Materials

S1.1. X-ray data collection, processing and structure determination

The PROXIMA I beamline at Soleil, with an ADSC Q315 3x3 CCD detector, was used to collect a native dataset from one crystal to high resolution, using 0.98 Å wavelength X-rays. A dataset intended to record the anomalous scattering from the intrinsic Fe and Mn atoms was collected from a different crystal, with the X-ray wavelength set at 1.73 Å (giving f'' of 3.8e^- and 3.4e^-, respectively. Diffraction images were indexed and scaled using HKL2000 (1) in space group $P4_22_12$. PHENIX (2) was used to obtain initial phase information by SAD (single wavelength anomalous diffraction), using the anomalous scattering from the metal atoms. Six metal atoms were located, consistent with a trimer in the asymmetric unit, with an initial figure of merit of 0.21 and which subsequently refined to a final figure of merit of 0.81 and phase error of 25.2° at 2.5 Å resolution. PHENIX was able to build 1694 (95.8%) of the residues in the three identical subunits automatically in the electron density map, along with 570 water molecules, to give a final model with an R_{work} of 20.0% (and R_{free} of 22.1%). This structure was then used for molecular replacment for the higher resolution (1.65 Å) native dataset, using MOLREP (3). Subsequent refinement and model building were performed using REFMAC (4) and COOT (5). Each of the subunits, consisting of 589 amino acids, was modelled in full except for the first 18-21 residues at the N-terminal ends, where the electron density was too weak or disordered. The three subunits are identical, with rmsds of 0.24 - 0.44 Å for 569 aligned residues and average main chain B-factors of 17.1 Å^2, 18.1 Å^2 and 21.5 Å^2. The metal atom B-factors are in the range 20 ± 2 Å^2 for Fe and 16 ± 2 Å^2 for Mn. A molecule of phosphate is bound to each of the active sites in a bridging position between the Fe and Mn atoms, with B-factors of 21.2 Å^2, 22.8 Å^2 and 25.8 Å^2. Five N-acetyl glucosamine sugar residues were also modelled in each subunit, with an average B-factor of 45.8 Å^2. Validation of the final model was performed using MOLPROBITY (6). The processing and refinement statistics are summarised in table 1.

S1.2. Small angle X-ray scattering analysis

Small angle X-ray scattering (SAXS) data for PPD1 (1-4 mg/mL concentration) were collected using the SWING beamline at the SOLEIL synchrotron with 1.03 Å X-rays and a sample-to-detector distance of 207.5 cm. Data were recorded from 1 s exposures on an in-vacuum AVIEX 170 × 170

mm^2 CCD detector over an angular range corresponding to momentum transfer q = 0.005 - 0.5 $Å^{-1}$ (q = 4π(sinθ)/λ, where 2θ is the scattering angle). Buffer scans were subtracted from corresponding PPD1 exposures. Data averaging and reduction were carried out using SOLEIL's FOXTROT suite. A Guinier approximation was performed with PRIMUS (7) over the region $qR_g < 1$, where R_g is the radius of gyration. Profile regularisation and the distance distribution function were obtained with GNOM (8). An experimental fit using the hexamer crystal structure was performed using CRYSOL and *ab initio* 3D shape reconstructions were performed by programs DAMMIF (to 0.16 $Å^{-1}$) and GASBOR (to 0.44 $Å^{-1}$), the latter also using the EMBL Hamburg ATSAS web server (9).

Figure S1 Small angle X-ray scattering (SAXS) of PPD1. (a) An *ab initio* shape reconstruction by DAMMIF using $P3_2$ symmetry, showing consistency between the predicted molecular shape (grey beads) and the hexameric construct based on the crystal structure (red cartoon). Water molecules have been omitted. (b) Experimental Kratky plot (Intensity x q^2 vs q), showing that the protein is predominantly folded under the conditions of the SAXS experiment.

(a)

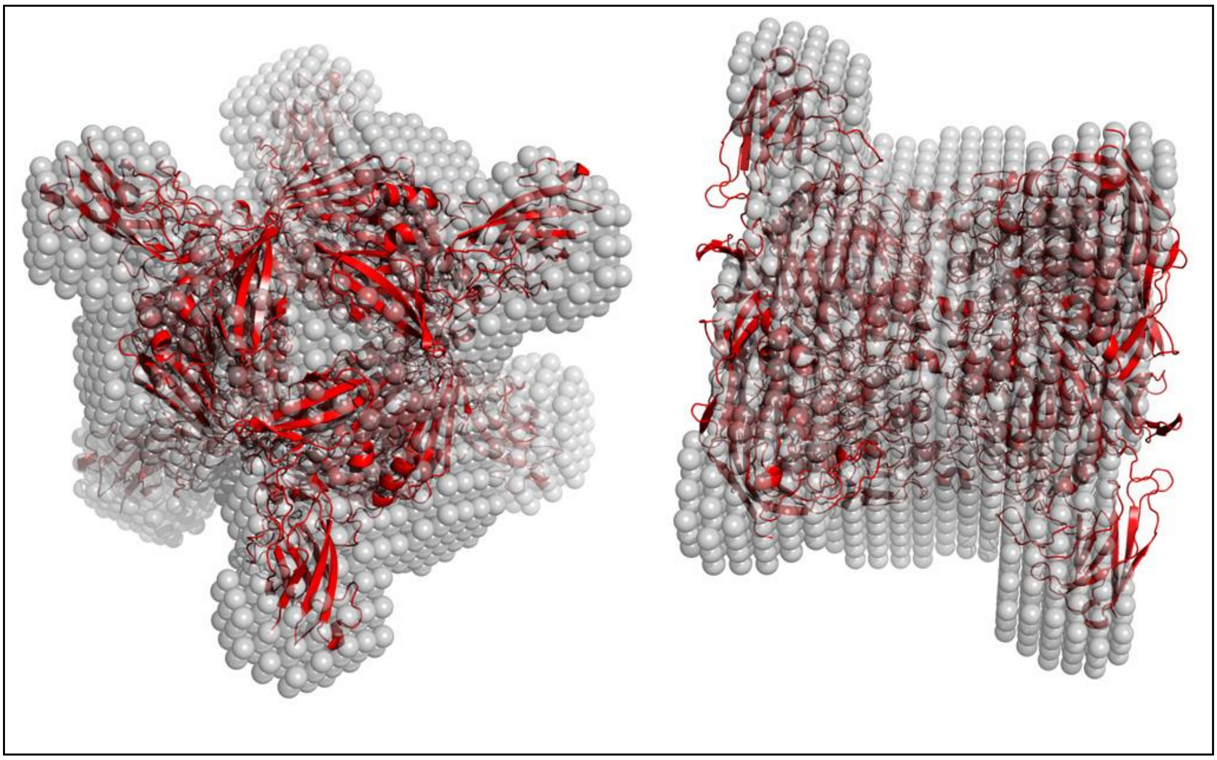

(b)

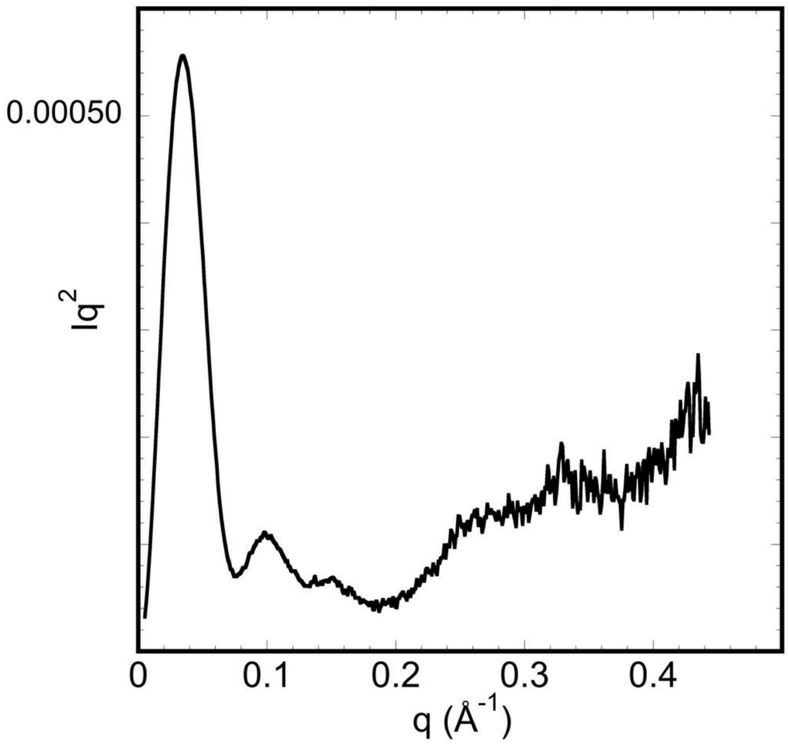

Figure S2 Anomalous scattering difference maps for

PPD1 obtained from two different crystals. The maps are calculated at the 7.5σ level for diffraction data measured at wavelengths of 1.74 Å (a) and 0.98 Å (b). Fe and Mn atoms are shown as purple and pink spheres respectively. The difference densities are consistent with the presence of Mn and Fe atoms at the active site, showing the increase of the anomalous signal expected from Fe in changing the x-ray wavelength, and the similar level of anomalous scattering expected from Fe and Mn at the shorter wavelength.

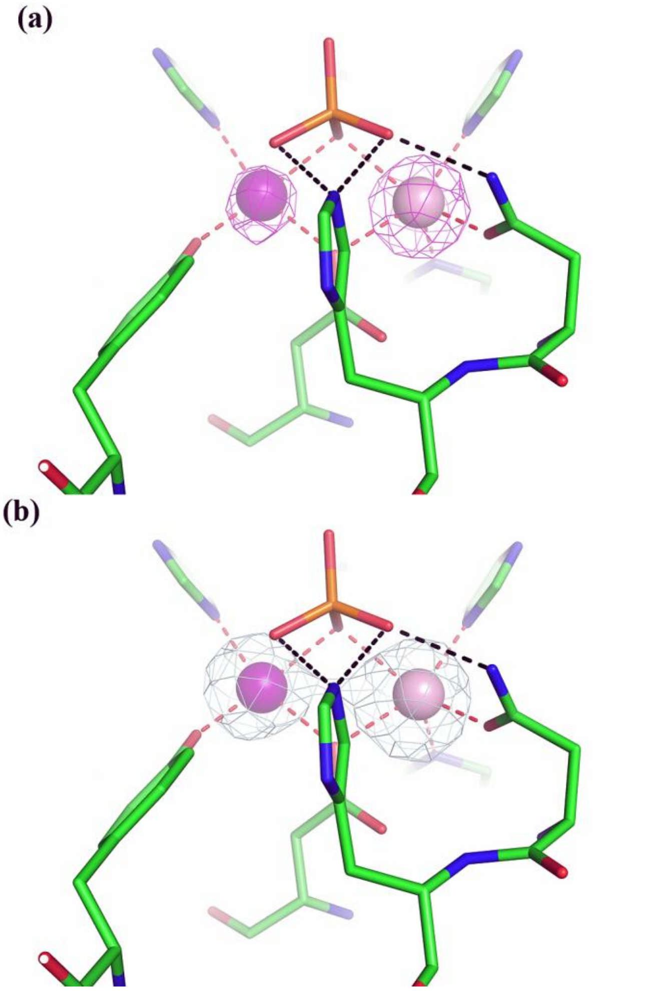

Figure S3 The distinctness of the new FN3 N-teminal domain (1-150, green) of PPD1 is evident by comparison (using SSM alignment) to residues 1-120 in the amino acid sequence of sweet potato PAP (1xzw, red), which contains the previously assigned FN3 domain for this PAP (and a similar result is obtained for comparison to red kidney bean PAP).

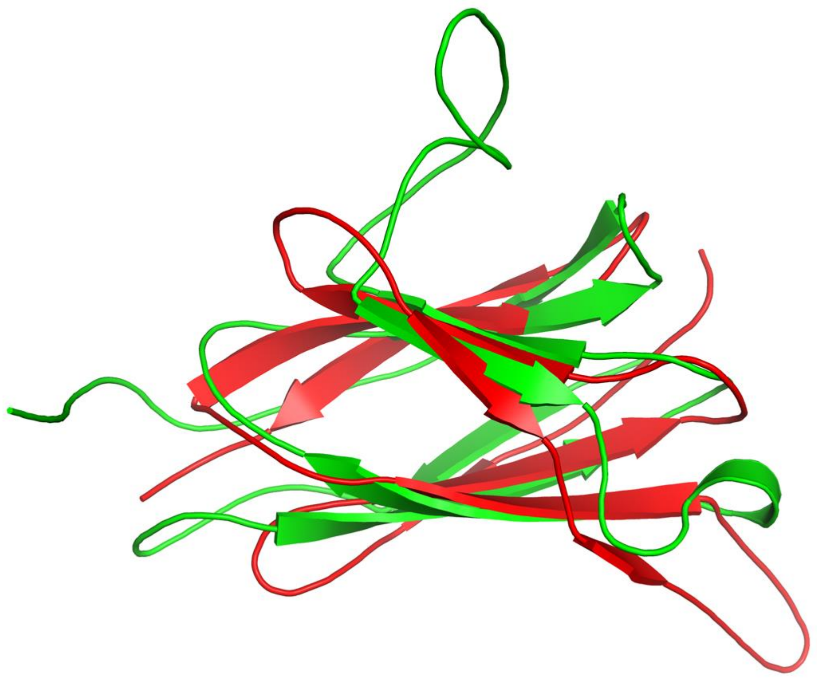

Table S1 Data Collection and Refinement Statistics

Resolution limits (Å)	48.8 – 1.65
(last shell)	(1.71-1.65)
No. recorded reflections	6613937
No. unique reflections	332100
Completeness (%) (last shell)	98.6 (99.8)
Multiplicity (last shell)	6.7 (6.6)
I / σ(I) (last shell)	19.0 (2.0)
Rmerge (%)	9.3 (70.0)
Wilson B-factor (Å^2)	18.6
No. protein atoms	15294
No. solvent atoms	2818
No. of sugars	15
Rcryst (%)	15.8
Rfree (%)	18.4
ML based ESU (Å)	0.051
Baverage main-chain (Å^2)	18.9
r.m.s. deviations:	
bond length (Å)	0.017
bond angle (°)	1.651

$R_{merge} = \Sigma|I_{hkl}-<I_{hkl}>|/\Sigma I_{hkl}$

$R_{cryst}=\Sigma|F_{obs}-F_{calc}|/\Sigma F_{obs}$

R_{free} : R-factor using a subset of 5% of random reflections excluded from refinement.

ML ESU: Maximum likelihood estimated standard uncertainty in REFMAC. Values in parentheses refer to the outermost resolution shell

References

1. Otwinowski Z & Minor W (1997) Processing of X-ray Diffraction Data Collected in Oscillation Mode. *Macromolecular Crystallography, part A*, Methods of Enzymology, eds Carter CW & Sweet RM (Academic Press, New York), Vol 276, pp 307-326.
2. Adams PD, *et al.* (2010) PHENIX: a comprehensive Python-based system for macromolecular structure solution. *Acta Cryst D*66:213-221.
3. Vagin A & Teplyakov A (1997) MOLREP: an automated program for molecular replacement. *J Appl Cryst* 30:1022-1025.
4. Murshudov GN, Vagin A, & Dodson EJ (1997) Refinement of macromolecular structures by the maximum-likelihood method. *Acta Cryst D* 53(3):240-255.
5. Emsley P & Cowtan K (2004) Coot: Model-building tools for molecular graphics. *Acta Cryst D*60(12 I):2126-2132.
6. Davis IW, *et al.* (2007) MolProbity: all-atom contacts and structure validation for proteins and nucleic acids. *Nucl Acid Res* 35(Web Server Issue):W375-W383.
7. Konarev PV, Volkov VV, Sokolova AV, Koch MHJ, & Svergun DI (2003) PRIMUS: a Windows PC-based system for small-angle scattering data analysis. *J Appl Cryst* 36:1277–1282.
8. Svergun DI (1992) Determination of the regularization parameter in indirect-transform methods using perceptual criteria. *J Appl Cryst* 25:495–503.
9. Petoukhov MV, Konarev PV, Kikhney AG, & Svergun DI (2007) ATSAS 2.1 – towards automated and websupported small-angle scattering data analysis. *J Appl Cryst* 40:s223-s228.
